# Interaction between numbers and size during visual search

**DOI:** 10.1007/s00426-016-0771-4

**Published:** 2016-05-03

**Authors:** Florian Krause, Harold Bekkering, Jay Pratt, Oliver Lindemann

**Affiliations:** 1 Department of Cognitive Neuroscience, Maastricht University, Maastricht, The Netherlands; 2 Brain Innovation B.V., Research, Maastricht, The Netherlands; 3 Radboud University Nijmegen, Donders Institute for Brain, Cognition and Behaviour, Nijmegen, The Netherlands; 4 Department of Psychology, University of Toronto, Toronto, Canada; 5 Division of Cognitive Science, University of Potsdam, Potsdam, Germany

## Abstract

The current study investigates an interaction between numbers and physical size (i.e. size congruity) in visual search. In three experiments, participants had to detect a physically large (or small) target item among physically small (or large) distractors in a search task comprising single-digit numbers. The relative numerical size of the digits was varied, such that the target item was either among the numerically large or small numbers in the search display and the relation between numerical and physical size was either congruent or incongruent. Perceptual differences of the stimuli were controlled by a condition in which participants had to search for a differently coloured target item with the same physical size and by the usage of LCD-style numbers that were matched in visual similarity by shape transformations. The results of all three experiments consistently revealed that detecting a physically large target item is significantly faster when the numerical size of the target item is large as well (congruent), compared to when it is small (incongruent). This novel finding of a size congruity effect in visual search demonstrates an interaction between numerical and physical size in an experimental setting beyond typically used binary comparison tasks, and provides important new evidence for the notion of shared cognitive codes for numbers and sensorimotor magnitudes. Theoretical consequences for recent models on attention, magnitude representation and their interactions are discussed.

## Introduction

In our modern society, dealing with numbers has become an integral part of our daily life. It is therefore important to understand how our brains process the numerical information that surrounds us. Several authors have recently suggested that the cognitive representation of numbers shares common codes with representations of size-related information from sensorimotor processes (Walsh, 2003, 2015; Andres, Olivier, & Badets, 2008; Lindemann & Fischer, 2015). Evidence supporting this idea of a generalised magnitude system can be found in a variety of behavioural studies showing that a magnitude comparison in one domain is influenced by magnitude information in another, task-irrelevant domain. For instance, effects of cognitive interference have been observed between the processing of numerical size and the perception of physical size (Besner & Coltheart, 1979; Henik & Tzelgov, 1982), luminance (Cohen Kadosh & Henik, 2006) as well as perceived affordances of objects (Badets, Andres, Di Luca, & Pesenti, 2007) or the amount of tactilely stimulated fingers (Krause, Bekkering, & Lindemann, 2013). In addition, numbers have been shown to influence motor control, such as the planning of the finger aperture while grasping (e.g. Lindemann, Abolafia, Girardi, & Bekkering, 2007; Moretto & di Pellegrino, 2008) or the required motor force (Vierck & Kiesel, 2010; Krause, Lindemann, Toni, & Bekkering, 2014).

The most often replicated finding suggesting shared magnitude representations for numbers and visual perception is the so-called size congruity effect (Henik & Tzelgov, 1982). In a typical size congruity study, participants are presented with two digits that differ in numerical as well as physical size (e.g. ) and are asked to indicate which one is physically larger by pressing one of two buttons. Under these conditions, reaction times are shorter if the physically larger stimulus is also the numerically larger one (e.g. ), as compared to the situation in which the physical and numerical size of the stimuli are incongruent (e.g. ; Besner & Coltheart, 1979; Henik & Tzelgov, 1982; Pansky & Algom, 1999).

Importantly, at the core of the size congruity paradigm lies the explicit comparison between two stimuli. Recently, several authors have argued that the observed apparent interaction between numerical and physical size relies on specifics of the experimental set-up of such a binary comparison (e.g. Risko, Maloney, & Fugelsang, 2013; Santens & Verguts, 2011). Risko et al. (2013) reasoned that in the classical size congruity paradigm with two presented digits, the physically larger digit automatically captures attention and is hence processed before the other digit, leading to a temporal congruity effect: if the first processed digit in a magnitude comparison task is the numerically larger one, reaction times are known to be faster than when the first processed digit is the numerically smaller one (Schwarz & Stein, 1998). While size congruity effects have also been reported for a variation on the classical paradigm, in which a single digit was presented and had to be explicitly compared to a pre-defined standard (Schwarz & Heinze, 1998; Schwarz & Ischebeck, 2003), this variation is still based on an explicit comparison between two entities. Santens and Verguts (2011) have argued that in such an explicit comparison between two numbers, where two motor responses can be given (one corresponding to each number), congruity can be solely defined relative to the binary comparison at hand: if one of the numbers is physically larger and also numerically larger, both the task-relevant physical and the task-irrelevant numerical magnitude will—due to the instructed mapping of magnitude and response—activate the same motor response, leading to a shorter response time than in a situation where one number is numerically larger but the other one is physically larger, such that the task-relevant physical and task-irrelevant numerical magnitude activate opposing motor responses.

Given these task-specific explanations of size congruity, the question arises whether an interaction between numerical and physical size can also occur in an experimental setting beyond binary comparison tasks, where congruity cannot be defined solely in terms of these specifics. The demonstration of a size congruity effect in a task that is experimentally and cognitively different from the previously used binary comparison tasks would suggest a more general interaction mechanism and could provide new evidence for the notion that size-related information from different domains is represented by a generalised magnitude system (Walsh, 2003, 2015). The present study therefore investigates whether an interaction of numerical and physical size in form of a size congruity effect does also emerge during a visual feature search for a target stimulus among many simultaneously presented distractor stimuli.

Accumulating evidence for an early impact of number meaning on visual attention and perception makes a possible existence of a size congruity effect in visual search plausible. For instance, Corbett, Oriet and Rensink (2006) demonstrated that the cognitive system is capable of rapidly extracting numerical information from briefly presented visual displays such that the visual comparison of two sets of Arabic digits could be made more efficiently than the comparison of sets of letters or meaningless control stimuli. Moreover, effects of numerical information on visual attention have been reported by Fischer, Castel, Dodd and Pratt (2003; but see also Ranzini, Dehaene, Piazza, & Hubbard, 2009; Bonato, Priftis, Marenzi, & Zorzi, 2009). The authors employed a simple visual detection task and showed that the mere presentation of digits has an impact on the likelihood to detect laterally presented visual targets depending on the numerical size of the digit; that is, consistent with the spatial arrangement of numbers along a hypothetical mental number line (Dehaene, Bossini, & Geraux, 1993), small numbers (digits 1 and 2) caused a shift in visual attention to the left and large numbers (digits 8 and 9) to the right side of space. This finding shows that the mere perception of Arabic digits results in an activation of analogue magnitude representations.

Furthermore, Schwarz and Eiselt (2012) recently demonstrated that the magnitude information of different simultaneously presented Arabic digits becomes automatically activated and affects the visual search for a target number in these displays. The authors required their participants to find a target digit among distractor digits in displays in which the average numerical distance between the target and distractors was systematically varied. Their analyses of the visual search performance revealed that the speed and accuracy under these conditions increased linearly with the numerical distance. The authors interpret this as evidence that the automatically activated numerical representation supports the classification of visual stimuli as targets and distractors. This indicates that the perception of arrays of Arabic digits simultaneously activates multiple magnitude representations associated with different digits in a display. Interestingly, it has recently been demonstrated that the impact of numerical information on visual search performance can even be observed if the semantic and visual similarity between target and distractor items is varied simultaneously (Godwin, Hout, & Menneer, 2014). Even though these studies show that multiple numerical magnitude representations become automatically activated when searching for a target digit among distractor digits, it remains unclear at this point whether the numerical representations interfere with the processing of size-related visual stimulus features during visual search.

The current study further investigates the impact of numerical information on visual search performance and asks whether the availability of numerical magnitude information does selectively affect the processing of physical size during a feature search. To be precise, we presented a set of digits and asked participants to find the item that was physically larger (or smaller) than the other items. The congruity between physical size and the task-irrelevant numerical size of the target item was systematically varied. Since the intention to search for an item of a particular physical size should lead to more attentional capture of all objects carrying this feature (e.g. Proulx & Egeth, 2008; Kiss & Eimer, 2011), it seems plausible to assume that if physical size interacts with numerical size, a size congruity effect during visual search should be observed.

## Experiment 1

The goal of this experiment was to explore the interaction between the processing of numerical size and physical size in a visual search task. In a new paradigm, we presented a set of 8 or 18 different digits, with the target digit deviating from the distractor digits in physical size. A size congruity effect during visual search was expected; that is, the time it takes to detect a physically large target among physically smaller distractors should be faster when the task-irrelevant numerical size of the target is large, and vice versa for physically small targets.

### Method

#### Participants

Nineteen students of Radboud University Nijmegen (15 females) between 18 and 26 years of age (mean = 20.89, SD = 2.21) participated in the study in return of credit points or 5 Euro. All of them reported to have normal or corrected-to-normal vision.

#### Set-up and material

Participants were seated in front of a table with a built-in horizontally oriented touch-sensitive computer screen and a custom-made response button (distance between response button and screen centre: 21 cm). The viewing distance was approximately 60 cm. Releasing the response button recorded the detection of a target. The experiment was controlled using the software *Expyriment* (Krause & Lindemann, 2014).

**Fig. 1:**
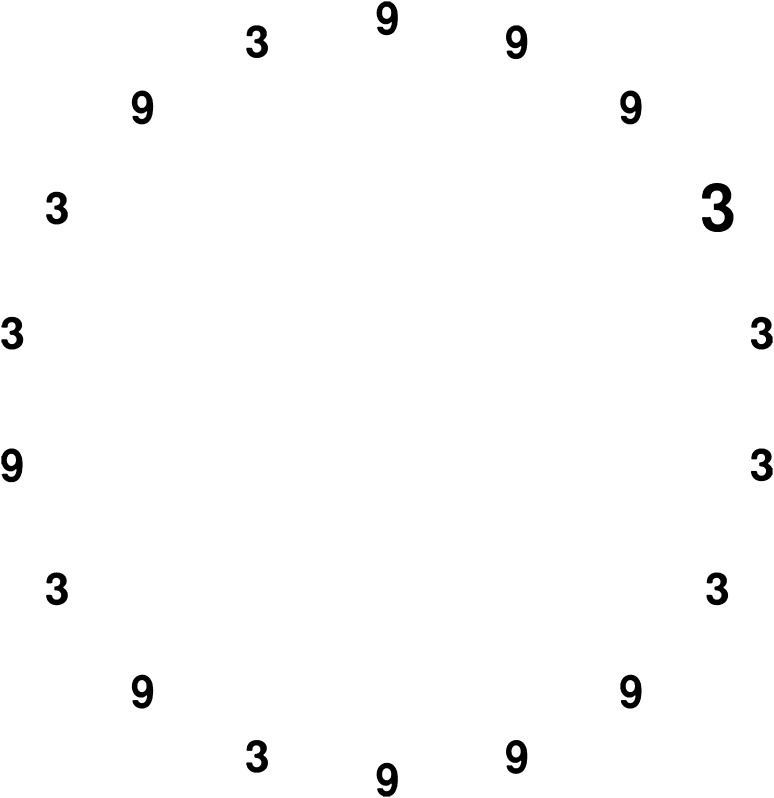
Illustration of an incongruent trial in a search set with 18 items (large search set size). Stimuli in the experiments were presented in grey colour on a black background

All stimuli in the visual search task consisted of the Arabic digits ‘2’, ‘3’, ‘8’ and ‘9’ printed in grey colour (photometric luminance: 62.01 cd/m^2^) on a black background (photometric luminance: 0.75 cd/m^2^) using a sans serif font type (see Fig. 1 for an illustration). Search sets comprised either 8 (small set size) or 18 (large set size) items arranged randomly, but equally spaced, in a circle with a visual angle of 19.85°. The distractor digits subtended a vertical visual angle of 0.57° and a horizontal visual angle of 0.38°. The target deviated from the distractors in physical size (larger: vertical visual angle of 0.86°, horizontal visual angle of 0.57°; smaller: vertical visual angle of 0.28°, horizontal visual angle of 0.19°).

Half of the items in each set were instances of one numerically small digit (‘2’ or ‘3’). The other items comprised instances of one numerically large digit (‘8’ or ‘9’). Two different sets of digits were used (‘2’–‘8’ and ‘3’–’9’).

#### Procedure

The experiment comprised two different blocks in which the target item was either defined as being (1) physically smaller or (2) physically larger, compared to the distractors. Participants received a verbal as well as written description of the experiment and were informed before each block about the next target type.

Each trial started with the presentation of a central fixation cross. After the participants depressed the response button, a blank screen was presented for a random duration between 1000 and 2000 ms, followed by the display of the circular search set. Participants were instructed to search for the target item. As soon as the target was detected, participants had to release the response button and point to the target location. A button release in the first 200 ms was considered to be an anticipatory response, and a button release later than 1500 ms was considered to be a too slow response (based on average search times from previous pilot data). To ensure that the target was found before response initiation, all items in the search set were masked at the moment of response button release.

#### Design

The order of experimental blocks (physically small target, physically large target) was counterbalanced across participants. Each block comprised 160 trials consisting of all possible combinations of the two different digit set types (‘2’–’8’, ‘3’–’9’), the two set sizes (small: 8 items, large: 18 items) and the two relative numerical sizes of the target (small, large). The order of trials was randomised. The total duration of the experiment was approximately 20 min.

### Results

One participant stopped the experiment prematurely and was excluded from further statistical analysis. The remaining participants made few errors and identified the target incorrectly or responded anticipatorily (i.e. search times shorter than 200 ms) in less than 1 % of the trials. In 2.90 % of all trials, the target was detected too slowly (i.e. search times greater than 1500 ms). Incorrect, anticipatory and slow trials were removed from further response time analyses.

**Fig. 2:**
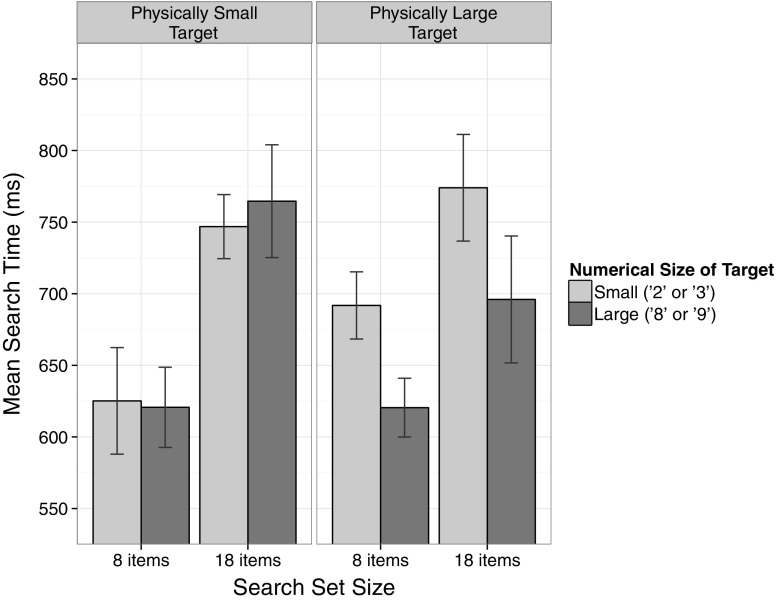
Mean search times for each task in Experiment 1. When searching for the physically larger target item, search times were shorter when the target items was numerically large as well, compared to when it was numerically small. *Error bars* represent 95 % confidence intervals for within-subject designs (Morey, 2008)

Average search times, defined as the median duration between search set onset and response button release, were calculated for each participant and condition and were submitted to a 2 × 2 × 2 analysis of variance (ANOVA) with the within-subject factors Physical Size of Target (small, large), Numerical Size of Target (small, large) and Search Set Size (8 items, 18 items). Figure 2 depicts the mean search times as a function of all three factors. The ANOVA revealed a main effect of Search Set Size, *F*(1, 17) = 197.09, MSE = 2045.85, *p* < 0.001, *η*_p_^2^ = 0.92 (7 distractors = 640 ms; 17 distractors = 745 ms), reflecting the classical phenomenon that targets are detected faster among fewer distractors (e.g. Sagi & Julesz, 1987). There was furthermore a significant main effect of Numerical Size of Target, *F*(1, 17) = 50.17, MSE = 831.58, *p* < 0.001, *η*_p_^2^ = 0.75. Search times were shorter when the target item was numerically large (675 ms) compared to when it was numerically small (709 ms). The interaction between Physical Size of Target and Search Set Size was significant as well, *F*(1, 17) = 12.50, MSE = 2094.76, *p* < 0.01, *η*_p_^2^ = 0.42; that is, the effect of Physical Size of Target was stronger for search sets with 8 items, *F*(1, 17) = 2.36, MSE = 8392.58, *p* = 0.14, *η*_p_^2^ = 0.12, compared to search sets with 18 items, *F*(1, 17) = 0.61, MSE = 12,739.26, *p* = 0.45, *η*_p_^2^ = 0.03.

Importantly, in line with our hypothesis, there was a significant interaction between Physical Size of Target and Numerical Size of Target, *F*(1, 17) = 24.11, MSE = 2467.95, *p* < 0.001, *η*_p_^2^ = 0.59. For physically large targets, search times were significantly shorter, when the target was numerically large (658 ms), compared to when the target was numerically small (733 ms), *t*(17) = 6.32, *p* < 0.001, *d* = 1.49, reflecting a size congruity effect. Interestingly, the reversed pattern for physically small targets (faster search times if the target is numerically small as well) was not statistically reliable (numerically small: 686 ms numerically large: 693 ms), *t*(17) = 1.00, *p* = 0.33, *d* = 0.24. No further effects were observed.

It is worth noticing that the data showed no trace of the well-known association between numerical size and spatial positions (e.g. Dehaene et al., 1993); that is, an additional analysis showed no difference between search times for targets with a horizontal position congruent to its numerical value (i.e. a numerically small target on the left side or a numerically large target on the right side; 683 ms) compared to incongruent ones (i.e. a numerically large target on the left side or a numerically small target on the right side; 677 ms), *t*(17) = 1.31, *p* = 0.21. Looking at lateral positions only (i.e. distance to centre of the display larger than 50 % of the radius of the circular search array) yielded comparable results, *t*(17) = 0.67, *p* = 0.51).

### Discussion

Consistent with our hypothesis, a congruity effect between the physical and numerical size of the target was found. The time it took to detect a physically large target was shorter when the numerical size of the target was large as well, compared to when it was small. The visual search size congruity effect was only present when participants were searching for a physically large item among physically small distractors. When the target digit was the physically smallest item in the search set, no interaction between numerical and physical magnitudes was observed. Given the design of the experiment, at least two factors could have led to this dissociation between the effects of the two physical size conditions: (a) the absolute size of the physically small targets could have been too small and their semantic value could not be processed, and (b) the stimuli used for the large digits (‘8’ and ‘9’) might be easier to detect in general. To control for these aspects, a second experiment was conducted.

## Experiment 2

To ensure that the absence of a reversed effect when searching for a physically small target found in the first experiment was not an artefact of the design, the goal of the present experiment was to examine the effects of numerical magnitude on the visual search performance as found in Experiment 1 with two modifications: (a) the overall stimulus size was increased by 100 % to ensure legibility, (b) a control condition was included in which the target item was indicated by a change in colour, while the physical size was the same as that of the distractor items. Moreover, to exclude that the dissociation of effects observed in Experiment 1 was a result of too low statistical power, the sample size was increased.

### Method

#### Participants

Thirty students of Radboud University Nijmegen (26 females) between 17 and 27 years of age (mean = 19.83, SD = 2.44) participated in the study in return of credit points or 5 Euro. All of them reported to have normal or corrected-to-normal vision.

#### Set-up and material

The stimuli and material used in this experiment were identical to the ones used in Experiment 1 with minor modifications. The distractor digits subtended a vertical visual angle of 1.15° and a horizontal visual angle of 0.76°. The target deviated from the distractors in either physically size (larger: vertical visual angle of 1.72°, horizontal visual angle of 1.15°; smaller: vertical visual angle of 0.57°, horizontal visual angle of 0.38°). In addition to being physically larger or smaller than the distractors, the target item could also be depicted in a light brown colour (photometric luminance: 54.82 cd/m^2^), but in the same size as the distractors. Furthermore, pairing each small digit with each large digit resulted in four different sets of digits (i.e. ‘2’–’8’, ‘2’–’9’, ‘3’–’9’, ‘3’–’8’).

#### Procedure and design

Each block comprised 160 trials consisting of all possible combinations of the four different digit set types (‘2’–’8’, ‘2’–’9’, ‘3’–’9’, ‘3’–’8’), the two set sizes (small: 8 items, large: 18 items) and the two relative numerical sizes of the target (small, large). The order of trials was randomised. The total duration of the experiment was approximately 30 min.

### Results

Trials with incorrect (<1 %), anticipatory (<1 %) and slow responses (2.51 %) were removed from the response time analyses.

**Fig. 3:**
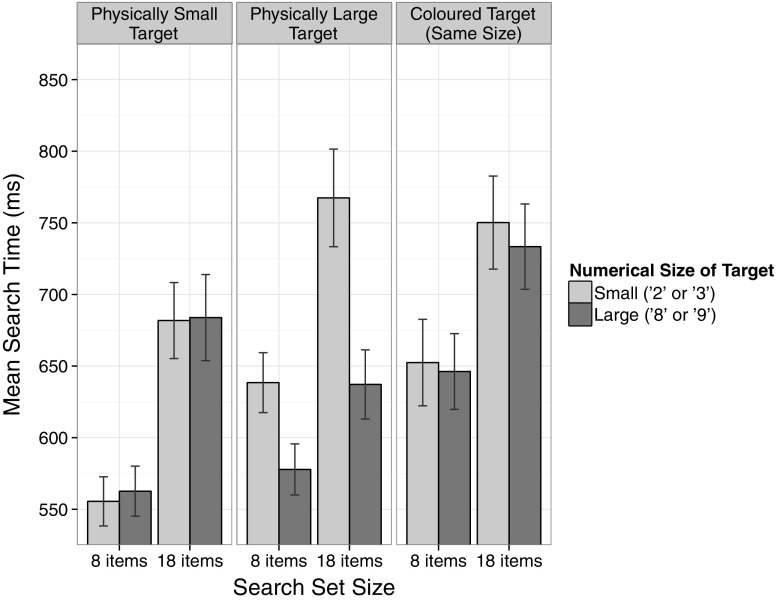
Mean search times for each task in Experiment 2. With a doubled stimulus size, the addition of a control condition, and a larger sample size, the findings of Experiment 1 were replicated. *Error bars* represent 95 % confidence intervals for within-subject designs (Morey, 2008)

Average search times, defined as the median duration between search set onset and response button release, were calculated for each participant and condition and were submitted to a 3 × 2 × 2 ANOVA with the within-subject factors Physical Size of Target (small, large, same), Numerical Size of Target (small, large) and Search Set Size (8 items, 18 items). Figure 3 depicts the mean search times as a function of all three factors. The ANOVA revealed a main effect of Search Set Size, *F*(1, 29) = 263.07, MSE = 3661.57, *p* < 0.001, *η*_p_^2^ = 0.90 (7 distractors = 606 ms; 17 distractors = 709 ms), reflecting that targets are detected faster among fewer distractors. There was also a significant main effect of Physical Size of Target, *F*(2, 58) = 9.02, MSE = 18,551.91, *p* < 0.001, *η*_p_^2^ = 0.24. Search times were faster for physically small targets (621 ms) compared to both physically large targets (655 ms), *t*(29) = −2.66, *p* < 0.05, *d* = 0.49, and coloured targets of the same size (696 ms), *t*(29) = −3.90, *p* < 0.01, *d* = 0.71. Furthermore, the effect of Numerical Size of Target reached significance, *F*(1, 29) = 57.96, MSE = 1807.05, *p* < 0.001, *η*_p_^2^ = 0.67, with numerically large targets (640 ms) being detected faster than numerically small targets (674 ms). There was also a significant interaction between Numerical Size of Target and Search Set Size, *F*(1, 29) = 13.00, MSE = 1396.15, *p* < 0.01, *η*_p_^2^ = 0.31; that is, the effect of Numerical Size of Target was stronger for search sets with 18 items, *F*(1, 29) = 47.03, MSE = 2234.08, *p* < 0.001, *η*_p_^2^ = 0.62, compared to search sets with 8 items, *F*(1, 29) = 18.41, MSE = 969.12, *p* < 0.001, *η*_p_^2^ = 0.39.

Crucially, consistent with our earlier results, there was a significant interaction between Physical Size of Target and Numerical Size of Target, *F*(2, 58) = 40.06, MSE = 2158.07, *p* < 0.001, *η*_p_^2^ = 0.58. For physically large targets, search times were significantly shorter, when the target was numerically large (608 ms), compared to when the target was numerically small (703 ms), *t*(29) = 10.75, *p* < 0.001, *d* = 1.96, reflecting a size congruity effect. Once again, the reversed pattern for physically small targets (faster search times if the target is numerically small as well) was not statistically reliable (numerically small: 619 ms numerically large: 623 ms), |*t*(29)| < 1. There was no effect of numerical size for targets with unchanged physical size (numerically small: 701 ms; numerically large: 690 ms), *t*(29) = 1.40, *p* = 0.17, indicating that the size congruity effect is not due to generally faster search times for numerically large targets. Furthermore, there was a significant three-way interaction between Physical Size of Target, Numerical Size of Target and Search Set Size, *F*(2,58) = 8.02, MSE = 1197.66, *p* < 0.01, *η*_p_^2^ = 0.22, reflecting that the interaction between physical and numerical size of the target was larger in search sets with 18 items, *F*(2, 58) = 33.54, MSE = 2288.97, *p* < 0.001, *η*_p_^2^ = 0.54, compared to search sets with 8 items, *F*(2, 58) = 18.08, MSE = 1066.76, *p* < 0.001, *η*_p_^2^ = 0.38. Finally, no congruency effect between the numerical size of the target and its horizontal position was observed in the overall search times (mean congruent = 635 ms; mean incongruent = 632 ms), *t*(29) = 0.53, *p* = 0.60 (lateral positions only: *t*(29) = 0.44, *p* = 0.66). No further effects were observed.

To obtain a clearer picture of the non-significant size congruity effect for the physically small target, congruent and incongruent trials of each participant were divided into six equally sized bins, with the first bin containing the shortest and the last bin containing the slowest search times. Then, a 6 × 2 repeated-measures ANOVA with the within-subject factors Time Bin (1, 2, 3, 4, 5, 6) and Congruency (congruent, incongruent) was performed. Besides a main effect of Time Bin, *F*(1.233, 35.745) = 306.17, MSE = 21,524.67, *p* < 0.001, *η*_p_^2^ = 0.91, the ANOVA revealed a significant interaction effect between Time Bin and Congruency, *F*(2.068, 59.986) = 3.71, MSE = 2191.74, *p* < 0.05, *η*_p_^2^ = 0.11, showing a difference in congruency effects across the time bins. A post hoc paired-samples *t* test (one-tailed) on congruency in the last time bin indicated a size congruity effect, *t*(29) = 1.91, *p* < 0.05, *d* = 0.35; that is, for long search times, finding the physically small target was significantly faster when the target was numerically small as well. Notably, when applying the same analysis to the control condition in which a differently coloured target of the same physical size had to be searched for, no significant interaction between Time Bin and Congruency was observed, *F*(2.33, 67.55) = 10.8, MSE = 2108.5, *p* = 0.35.

### Discussion

Experiment 2 replicated the results from the previous experiment using larger stimuli and an additional control condition. A congruity effect between the physical and numerical size of the target was again observed when searching for a physically large target. In the control condition where the target was differently coloured, but of equal physical size as the distractors, the target’s numerical value had no influence on search times. This is of particular importance, as it controls for general effects induced by perceptual differences between the stimuli, which were implicated by the significant main effect of numerical size. As in the first experiment, no congruency effect was observed when searching for a physically small target. Notably, the physical size of all stimuli has been doubled in the current experiment, compared to the previous one. Moreover, the statistical power of Experiment 2 has been increased substantially.

Obtaining the same pattern of results under these conditions suggests that it was not the absolute size of the target item in Experiment 1 that led to the observed asymmetry of the size congruity effect during visual search between the two physical size conditions. Interestingly, when searching for a physically small target, a congruity effect was present in the slowest search times, indicating a delayed interaction between numerical and physical size in this condition. Taken together, the results seem to imply a more general impaired semantic processing of physically small target items in the presence of relatively larger distractors (see also ‘General discussion’). Future research with a focus on this issue is needed to detail the specifics of this phenomenon.

More crucial for the current study, however, there might still be an alternative explanation for the size congruity effect observed in both experiments when searching for the physically large target. While the colour condition in Experiment 2 successfully controlled for a general facilitated processing of numerically large targets, it needs furthermore to be excluded that the observed effect is a result of local perceptual properties of the stimuli ‘8’ and ‘9’ (cf. Wong & Szücs, 2013), whose relevance in a feature search might scale with the physical size the stimuli are presented in. Such a difference in local perceptual properties compared to the other stimuli could theoretically explain a facilitation of detecting the numerically large stimuli when searching for the physically large target, while not finding the same facilitation for those stimuli in the other two conditions. To control for this potential confound, a third experiment was conducted.

## Experiment 3

To further control for the possible confound that the size congruity effect observed in Experiments 1 and 2 is merely a result of differences in local perceptual stimulus properties which get enhanced when the stimulus is enlarged, rather than being due to an interaction between the physical and numerical size of the target, an additional experiment aimed to replicate the finding of a size congruity effect in visual search using two different sets of LCD-style digits (as known from digital alarm clocks and watches). Importantly, the visual characteristics of LCD-style digits made it possible to construct stimulus sets in such a way that, while the semantic distance of the numerical values was systematically varied, they ensured minimal deviations of visual features between the physical symbols, by applying shape transformations (i.e. 180-degree rotation of a ‘6’ results in a ‘9’ and vertically mirroring a ‘2’ results in a ‘5’; see also Sobel, Puri, & Hogan, 2015). If the size congruity effect in visual search is indeed the result of an interaction between the physical and numerical size of the target, two predictions can be made: first, the size congruity effect in visual search should be replicated for LCD-style numbers and second, since semantic distance between numbers is known to affect a numerical size comparison between them, by varying the amount of representational overlap between the numbers (Moyer & Landauer, 1967; Dehaene, Dupoux, & Mehler, 1990), a congruity effect between numerical and physical size should be modulated by the semantic distance of the two different numerical sizes in the search array; that is, a larger size congruity effect should be observed for the stimulus set with the larger semantic distance between the two numerical sizes, due to the smaller amount of representational overlap. Importantly, participants only had to search for large targets and this change of the procedure implies that the congruity effect is not to be found in an interaction between Physical Size and Numerical Size, as in the preceding experiments, but instead is manifested in a main effect of Numerical Size.

### Method

#### Participants

Twenty students of Radboud University Nijmegen (18 female) between 17 and 25 years of age (mean = 20.45, SD = 2.14) participated in the study. All of them had normal or corrected-to-normal vision. Participants received credit points or 5 Euro for their participation.

#### Set-up and material

**Fig. 4:**
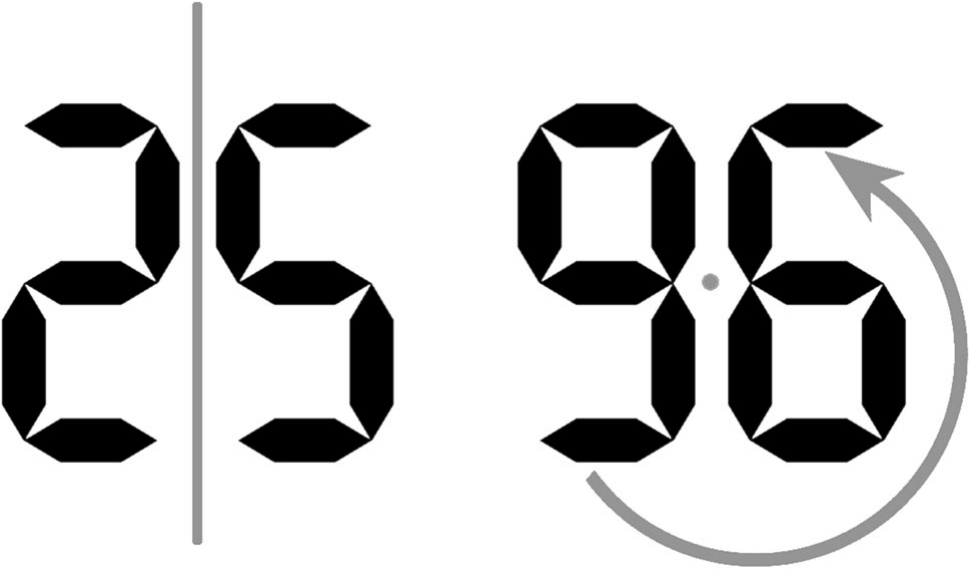
Illustration of matching the stimuli in each set in Experiment 3 for local perceptual features. The semantically close set contained LCD digits ‘5’ and ‘6’, while the semantically distant set contained LCD digits ‘2’ and ‘9’. Importantly, ‘2’ is identical to a vertically mirrored ‘5’ and ‘9’ is identical to a by 180° rotated ‘6’

The experimental set-up and material were identical to Experiment 2; merely the stimuli were modified. First, stimulus sets consisted of grey LCD digits (visual angle: horizontal = 0.57° or 0.86°, vertical = 1.15° or 1.72°; photometric luminance: 62.01 cd/m^2^). Second, the number stimuli were matched for maximal physical similarity, such that one set of numbers consisted of either a vertically mirrored or a by 180° rotated version of the symbols from the other set (see Sobel et al., 2015, for a previous application of this approach). The semantically distant set consisted of digits ‘2’ and ‘9’ and the semantically close set consisted of digits ‘5’ and ‘6’. See Fig. 4 for an illustration of the physical similarity matching.

#### Procedure and design

The procedure was the same as in Experiment 2, with the exception that displays with a physically small target were not realised and the only task was to search for the physically large item in each display. The experimental design consisted of one block, comprising 256 trials consisting of all possible combinations of the two different stimulus sets (semantically distant: ‘2’–’9’, semantically close: ‘5’–’6’), the two set sizes (small: 8 items, large: 18 items) and the two relative numerical sizes of the target (small, large). The order of trials was randomised. The total duration of the experiment was approximately 20 min.

### Results

Participants made erroneous responses (identifying an incorrect item to be the target) or responded faster than 200 ms in less than 1 % of all trials. Too slow detection of the target (i.e. search times larger 1500 ms) occurred in 6.35 %. These trials were removed from further response time analyses.

**Fig. 5:**
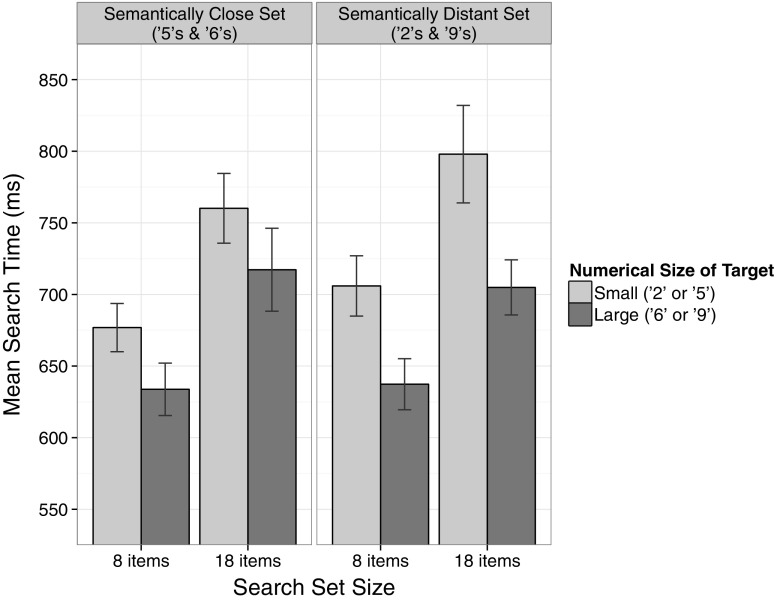
Mean search times in Experiment 3. The size congruity effect in visual search was larger for the semantically distant set, compared to the semantically close set. *Error bars* represent 95 % confidence intervals for within-subject designs (Morey, 2008)

A 2 × 2 × 2 ANOVA on the median search times with the within-subject factors Semantic Distance (distant, close), Numerical Size of Target (small, large) and Search Set Size (8, 18 items) was conducted. Figure 5 depicts the mean search times as a function of all three factors. As in the first two experiments, the analysis revealed an effect of the factor Search Set Size, *F*(1, 19) = 83.88, MSE = 3174.98, *p* < 0.001, *η*_p_^2^ = 0.82, with faster search times for small search sets with 8 items than for large search sets with 18 items (663 ms vs. 745 ms), as well as an effect of the factor Numerical Size of Target, *F*(1, 19) = 51.43, MSE = 2980.49, *p* < 0.001, *η*_p_^2^ = 0.73, reflecting that numerically large targets were detected faster than numerically small targets (673 ms vs. 735 ms). Furthermore, there was a significant main effect of Semantic Distance, *F*(1, 19) = 4.55, MSE = 1583.78, *p* < 0.05, *η*_p_^2^ = 0.19, reflecting that participants were faster to detect the target when the digits in the stimulus set were semantically close (697 ms), compared to when they were semantically distant (712 ms).

Importantly, the effect of Numerical Size of Target was modulated by Semantic Distance, *F*(1, 19) = 7.43, MSE = 1928.19, *p* < 0.05, *η*_p_^2^ = 0.28, as predicted by the notion that search time differences are driven by an interaction with semantic information. While numerically large targets were detected significantly faster than numerically small targets, this visual search size congruity effect was larger when the semantic distance between the target and the distractors was distant (671 ms vs. 752 ms), *t*(19) = 8.49, *p* < 0.001, *d* = 1.90, compared to a when the semantic distance was close (676 ms vs. 719 ms), *t*(19) = 3.46, *p* < 0.01, *d* = 0.77. No further effects were observed.

Finally, no congruency effect between the numerical size of the target and its horizontal position was observed in the overall search times (mean congruent = 680 ms; mean incongruent = 689 ms), *t*(19) = 1.06, *p* = 0.30 (lateral positions only: *t*(19) = 0.77, *p* = 0.45).

### Discussion

Experiment 3 replicated the size congruity effect in visual search for LCD-style numbers: participants were faster to detect the physically larger target when this target was one of the numerically large digits. Importantly, this visual search size congruity effect was modulated by the semantic distance between the numerical sizes of the presented items. In other words, the effect was stronger when the numerical sizes of the target and the distractors were semantically more distant (cf. Pansky & Algom, 1999 for a similar modulation in the classical size congruity paradigm). This modulation reassures us that the observed congruity effect is actually dependent on the processing of the numerical size information, rather than a mere facilitation of the physically enlarged target stimulus due to increased saliency.

## General discussion

The present study provides the first empirical evidence for an interaction between physical and numerical size during visual search; that is, targets with congruent physical and numerical size were detected faster compared to targets with an incongruent configuration. Perceptual differences of the stimuli that might account for the observed differences in search performance (cf. Wong & Szücs, 2013) were controlled by a condition in which the target item was defined by the colour and not by the size, showing no difference between the numerical stimuli (Experiments 2), and by demonstrating a modulation of semantic distance on the size congruity effect, using different search sets with LCD-like stimuli that were perceptually matched by mirroring and rotation (Experiment 3).

The observation of a size congruity effect during visual search provides a substantial advancement over previous number processing research by demonstrating that an interaction between numerical and physical size can also occur outside the experimental specifics of classical size congruity paradigms. To be more precise, classical size congruity paradigms are centred around an explicit comparison task; that is, participants have a binary choice of which of two presented digits is numerically larger (e.g. Besner & Coltheart, 1979; Henik & Tzelgov, 1982; Pansky & Algom, 1999) or whether a single presented digit is numerically larger than a pre-defined standard (e.g. Schwarz & Heinze, 1998; Schwarz & Ischebeck, 2003). Participants then indicate their decision by pressing one of two response buttons, each representing one of the two choice alternatives. Several authors have argued that the specifics of this experimental set-up might explain the observed apparent interaction between numerical and physical size (e.g. Risko, Maloney, & Fugelsang, 2013; Santens & Verguts, 2011). For instance, Risko et al. (2013) argued that in an explicit binary comparison task reaction times are subject to attentional capture effects which lead to a temporal congruity effect (Schwarz & Stein, 1998), rather than to a size congruity effect. Furthermore, Santens and Verguts (2011) pointed out that in a typical size congruity paradigm, congruity can be defined only relative to the comparison task at hand: if the right of two presented stimuli is physically larger and also numerically larger, both the task-relevant physical and the task-irrelevant numerical magnitude will activate a ‘right larger’ code, leading to a faster response than in a situation where only one magnitude is activating a ‘right larger’ code, while the other is activating a ‘left larger’ code. Given these task-specific explanations of the nature of the classical size congruity effect, a demonstration of a similar interaction between numerical and physical size in an experimental paradigm that does not entail an explicit comparison between two stimuli would indicate that size congruity is not limited to occur within the experimental constraints of a binary comparison task. The current study is an instance of such a demonstration, since the response latencies revealed a size congruity effect in a visual search task in which a target item had to be found in a display with many stimuli. Since the congruity effect in the current study was measured with the same responses—the release of the start button once the target had been detected—for both large and small targets, any explanation relying on response competition (cf. Santens & Verguts, 2011) cannot account for the present congruity effect.

Moreover, with the current visual search paradigm we exclude the presence of a temporal congruity effect (Schwarz & Stein, 1998) resulting from attentional capture (cf. Risko et al., 2013), since temporal congruity applies merely to situations in which two stimuli (one numerically small and one numerically large digit) are processed sequentially. Even if one supposes that items were processed strictly sequentially and that the target digit in the present paradigm was always processed first, the account of temporal congruity requires the additional assumption that participants stopped their visual search after they have processed the first distractor. First, this assumption of an early termination is in conflict with the set size effects found in all experiments. Second, even if the processing would sometimes be restricted to the target and one distractor, this distractor was in 43 to 47 % of the cases of the same numerical size as the target and could not induce any cognitive interference. We therefore consider this explanation for the observed size congruity effect as very unlikely.

The present findings are, however, in line with different recent general proposals which assume that attention and the coding of magnitude information are two mutually dependent processes (e.g. Risko et al., 2013; Fischer et al., 2003). While Fisher et al. (2003) argued that numerical size has an influence on spatial attention, Risko et al. (2013) further discuss a possible influence of attention on magnitude judgements. More specifically, they speculated that if different types of magnitude share a common code (Walsh, 2003), then ‘bias[ing] attention to one type of magnitude […] could produce a bias to attend to a similar dimension of other types of magnitude’ and ‘looking for larger objects might bias one to attend to large numbers’ (Risko et al., 2013, p. 1146). The present study now provides the first direct evidence for exactly this notion: directing attention to the physically larger item during a visual search seems to produce an unintentional bias to attend to the numerically large items as well. Importantly, this was not the case if the visual search was guided by stimulus features that are not size-related (e.g. colour), emphasising that the current finding represents an interaction between two sources of magnitude information. Moreover, magnitude interaction was enlarged when targets and distractors were numerically more distant, even if perceptual features were kept constant. Based on earlier findings which demonstrated that semantic distance between numbers affects a numerical size comparison between them (Moyer & Landauer, 1967; Dehaene et al., 1990), an enlarged congruity effect between numerical and physical size for the numerically more distant target and distractor items in the search array indicates that numerical size is being processed and affects the visual search. Together, these findings are reassuring us that the search time differences observed in the two experiments are not the consequence of an advantage of numerically larger target digits in visual search, but are indeed a result of an interaction between number meaning and physical size, that is, a size congruity effect in visual search. The current findings are therefore in line with the notion that numerical information is processed by a generalised magnitude system (Walsh, 2003) which originally emerged to serve perception and action.

While the inclusion of different search set sizes was initially motivated by gaining more insights over participants engagement in the experimental task (since larger search sets should lead to longer search times^1^; Sagi & Julesz, 1987), the observed interaction of the size congruity effect with the search set size in Experiment 2 is not in conflict with the notion of a generalised magnitude system. Since larger set sizes result in longer search times, the modulation of the size congruity effect is most parsimoniously explained by differences in processing times: longer processing times should lead to a deeper processing of the task-irrelevant numerical information and should therefore cause a stronger size congruity effect (see Schwarz & Ischebeck, 2003). Alternatively, one could argue that this finding is in line with studies showing that the amount of items in a display automatically activates numerical representations (Naparstek & Henik, 2010). Search set size could therefore be conceived as a third source of magnitude information in the visual search task, and it could be speculated that this third magnitude affects the processing of the two other magnitudes (physical size and numerical size). Any interaction between set size and numerical and physical magnitude information might hence be interpreted as an additional instance of within magnitude interference (cf. Walsh, 2003). However, the impact of search set size on the congruity effect as well as on physical size and numerical size did not manifest itself as a consistent pattern across the three experiments and we belief that future experimentation will be needed in order to better understand the underlying mechanisms.

The finding of a size congruity effect during visual search shows an impact of numerical size congruity on early visual attentional processes. As known from several studies on visual perception, top-down guidance of attention towards a certain stimulus feature (e.g. physical size) enhances the visual saliency of objects containing this feature (e.g. Wolfe, 1994; Proulx & Egeth, 2008; Kiss & Eimer, 2011). Recent evidence for the notion that numerical information guides visual search comes from Schwarz and Eiselt (2012), who demonstrated that the performance to find a target number among distractor digits is systematically influenced by the numerical distance between the target and the distractors. The current data extend this finding by showing that a visual search for a target that is solely defined by its physical size is also affected by task-irrelevant information about numerical size; that is, when attending to one particular type of physical target size (i.e. a physically large or a small target), the numerical size of the same target seems to guide attention as well, with faster search times if the numerical size matches the current target’s physical size. The role of non-visual stimulus features like semantic information in guiding spatial attention is still controversially discussed (Wolfe & Horowitz, 2004; Moores, Laiti, & Chelazzi, 2003; Belke, Humphreys, Watson, Meyer, & Telling, 2008). The present findings of size congruity in visual search might therefore additionally contribute to this ongoing debate in visual attention research by showing that semantic knowledge about number symbols under some conditions affects the performance in a visual feature search.

Importantly, in contrast to the vast majority of research on attentional effects of number processing (e.g. Fischer et al., 2003; Ranzini et al., 2009; Bonato et al., 2009), the present study examined the modulations of visual attention caused by non-spatial number features. It therefore provides empirical evidence for a number–perception interaction driven by a congruity between physical and numerical size of an item during visual search, irrespective of its spatial location. The additional analyses of spatial effects revealed that search performance in the present paradigm was not affected by spatial–numerical associations (see Hubbard et al., 2005 for a review), that is the congruency between the horizontal position of a target item and its numerical value. While earlier research showed that numerical size can bias attention to spatial positions (Fisher et al., 2003), the current finding suggests that participants do not use these spatial–numerical associations (e.g. Dehaene, 1992) to guide a visual search and that digits of different physical and numerical size are found equally well at all spatial positions.

To our surprise, a congruity effect between the physical and numerical size of the target was only observed when searching for a physically large target. An explanation for the lack of a size congruity effect in the small target condition is speculative at this point. One might assume that this finding reflects an impaired automatic processing of the target’s semantic meaning if the target digit is displayed in a small physical size. This weaker semantic activation of the number meaning could be due to the higher perceptual demands to process the detailed visual pattern of small symbols compared to large symbols. Alternatively, the impaired semantic processing of physically small targets might be driven by the fact that the feature search was performed significantly faster in this condition, compared to the colour and large target condition. Searching for the small target was thus perceptually easier and possibly more bottom-up driven by global visual stimulus features (e.g. total covered area or changes in luminance). In both cases, an impaired semantic processing would result in a delayed interaction between physical and numerical size. This notion receives empirical support by our data, since a size congruity effect could be found when looking only at the trials with the longest search times in Experiment 2. The replication of the same pattern of results with the doubled stimulus size (Experiment 2) further suggests that the impaired semantic processing of smaller stimuli is not the result of a too small absolute physical size. Instead, it seems that it was the relative size difference to the surrounding distractors which resulted in an impaired semantic processing of the relatively smaller target, possibly due to stimulus-driven attention to the many larger stimuli in the set (Proulx, 2010). This more general phenomenon might explain that the semantic information of targets was not processed to an extent that affected behaviour, if all distractor items were of larger size. Furthermore, in the design of the current experiments, the distance between the centre of each target and the centre of the distractors was fixed, leading to smaller distractor–target distances when the target was physically large, and larger distractor–target distances when the target was physically small. The resulting disproportionately in crowding could have also persistently undermined the congruity effect with the small targets (cf. Whitney & Levi, 2011). Taken together, it seems plausible to assume that semantic effects of number meaning predominately emerge in the present paradigm if the target is a larger symbol among smaller distractors. Future research with a specific focus on the effects of relative size differences between target and distractors on semantic processing in visual search is needed to better understand the details of the underlying cognitive mechanisms.

Eventually, the presented experiments give some new insights about the origin of behaviourally observed interactions between numerical and physical size (see, e.g. Schwarz & Heinze, 1998; Cohen Kadosh & Walsh, 2009; Santens & Verguts, 2011 for this debate). In general, two opposing accounts have been formulated: The first account holds that numerical and physical size interact at an early processing stage at which both stimulus features are coded into a common analogue magnitude representation (e.g. Schwarz & Heinze, 1998). Reaction time differences between congruent and incongruent configurations are thought to reflect a difference in the cognitive demand to create a common representation in the case that numerical and physical size are of different magnitude, compared to when they convey the same relative size (i.e. both small or both large). Empirical evidence for an interaction at an early representational stage comes from electrophysiological data, suggesting that the facilitations and interference in a size congruity task arise quickly with onsets well before 300 ms (Schwarz & Heinze, 1998; Szücs & Soltész, 2008).

The second account of the size congruity effect holds, however, that interference effects do not emerge at the level of magnitude representation. For instance, Cohen Kadosh and Walsh (2009) argue for a dual-code model of magnitude representation in which first, fast automatic representations are thought to be non-abstract and dependent on the notation or modality, while only later, slower intentional abstract representations might follow, depending on task and context. This notion of dual magnitude codes would not predict an interaction between physical and numerical size in early perception, because the automatic and unintentional processing of physical and numerical size is assumed to be initially based on independent representations. However, in contradiction to this prediction, the current study suggests the presence of an early interference effect as the presence of task-irrelevant numerical size automatically and unintentionally affected the detection of a target defined by its physical size. Furthermore, Santens and Verguts (2011) assume that, similar to Cohen Kadosh and Walsh (2009), different sources of magnitude information from different domains are represented entirely separately and interact only at later response-related stages of processing. The authors pointed out that the classical size congruity paradigm relies on a one-to-one mapping between the two choice alternatives (i.e. ‘left larger’, ‘right larger’) and the two motor responses (‘left’, ‘right’) and proposed a dual-route model, assuming a parallel processing of task-relevant and task-irrelevant stimulus dimensions of the digits that after a certain time results in a co-activation of both visual and numerical size information. In congruent trials, both size-related stimulus features activate the same response code, while in incongruent trials, the two stimulus dimensions map onto different response codes, resulting in a conflict at the level of response selection. This conflict is accompanied by longer response times. Recent evidence for such an explanation of the size congruity effect, which rejects the assumption of shared representations of numerical and physical size, comes from ERP and fMRI studies that suggest the presence of interference during response selection (e.g. Cohen Kadosh, Cohen Kadosh, Henik, & Linden, 2008; Szűcs & Soltész 2007).

In contrast to a classical size congruity paradigm, the visual search task used in the present study comprised several simultaneously presented digits and a single motor response (releasing the start button) to mark detection. While the finding of a size congruity effect in the longest search times only when searching for a physically small target (see ‘Experiment 2’) could in principle be interpreted as an indication for a relatively late effect, in our view, it is unlikely that the numerical and physical dimensions of each of the up to 18 digits in our experiments pre-activated up to 18 different motor responses, and hence more plausible that the observed cognitive conflict is not originating from response-related stages of processing. Notably, all experiments included an additional pointing response to the (masked) target position, and it can be argued that participants prepared the pointing response not as a second step, but as part of the initial response of releasing the start button. An interpretation of the results in terms of a conflict of pre-activated responses, however, assumes the rather unlikely parallel pre-activation of 8 (small search set) or even 18 (large search set) different responses. Furthermore, opposed to a classical size congruity paradigm, the task-irrelevant information of numerical size would not pre-activate one single response of these 8 or 18, but multiple ones (since half of the digits in the search display were of large numerical size and the other half was numerically small). The task-relevant information of physical size, on the other hand, would pre-activate exactly one response (since there was only one physically larger digit). This would lead to contradicting pre-activations in both congruent and incongruent situations. Given these substantial differences between the current visual search task and the classical size congruity paradigm, it is difficult to assume that the classical size congruity paradigm constitutes a visual search with only two items (a target and a distractor). Nevertheless, even if one interprets a classical size congruity paradigm this way, the current demonstration of a size congruity effect in a visual search with 8 and 18 items is a substantial extension of former findings, as it has been suggested that the processes underlying detection performance in small display sizes (e.g. 2 items close to each other as in the classical size congruity paradigm) differ from those underlying detection performance in larger displays (e.g. 18 items in a larger circular array as in the current study; see Meinecke & Donk, 2002).

Taken all together, the results of the present study seem to rather support an interaction between numerical and physical size on an early than on a late level. Eventually, more research will be needed to answer this question with sufficient certainty. For instance, an important open question the current study cannot answer is whether the interaction between numerical and physical size affects the initial allocation of attention or the stage of accepting or rejecting an item as the target in a serial process (see also Moores et al., 2003; Belke et al., 2008). The new visual search paradigm presented here, combined with eye-tracking techniques should stimulate future research in this direction.

## Conclusion

The current study is the first to observe an interaction between numbers and physical size (i.e. size congruity) during a visual feature search with multiple distractors. This novel finding demonstrates that interactions between numerical and physical size can occur beyond the experimental specifics of classically used binary comparison tasks, and provides important new evidence for the notion that numbers share cognitive codes with sensorimotor magnitudes (Walsh, 2003; 2015).

## Footnotes

1 Especially given the large radius of the circular search array used in the current study, since this effect is often even more pronounced in the periphery (Meinecke & Donk, 2002).
